# Thresholding Functional Connectivity Matrices to Recover the Topological Properties of Large-Scale Neuronal Networks

**DOI:** 10.3389/fnins.2021.705103

**Published:** 2021-08-16

**Authors:** Alessio Boschi, Martina Brofiga, Paolo Massobrio

**Affiliations:** ^1^Department of Informatics, Bioengineering, Robotics, Systems Engineering (DIBRIS), University of Genova, Genova, Italy; ^2^National Institute for Nuclear Physics (INFN), Genova, Italy

**Keywords:** connectivity matrix, functional connectivity, neuronal assemblies, threshold, topology, simulations

## Abstract

The identification of the organization principles on the basis of the brain connectivity can be performed in terms of structural (i.e., morphological), functional (i.e., statistical), or effective (i.e., causal) connectivity. If structural connectivity is based on the detection of the morphological (synaptically mediated) links among neurons, functional and effective relationships derive from the recording of the patterns of electrophysiological activity (e.g., spikes, local field potentials). Correlation or information theory-based algorithms are typical routes pursued to find statistical dependencies and to build a functional connectivity matrix. As long as the matrix collects the possible associations among the network nodes, each interaction between the neuron *i* and *j* is different from zero, even though there was no morphological, statistical or causal connection between them. Hence, it becomes essential to find and identify only the significant functional connections that are predictive of the structural ones. For this reason, a robust, fast, and automatized procedure should be implemented to discard the “noisy” connections. In this work, we present a Double Threshold (DDT) algorithm based on the definition of two statistical thresholds. The main goal is not to lose weak but significant links, whose arbitrary exclusion could generate functional networks with a too small number of connections and altered topological properties. The algorithm allows overcoming the limits of the simplest threshold-based methods in terms of precision and guaranteeing excellent computational performances compared to shuffling-based approaches. The presented DDT algorithm was compared with other methods proposed in the literature by using a benchmarking procedure based on synthetic data coming from the simulations of large-scale neuronal networks with different structural topologies.

## Introduction

The brain or more in general nervous systems are complex networks *par excellence*, made up of thousands of neurons synaptically interconnected. Such huge connectivity and the intrinsic topological organization make it possible to generate and integrate information from multiple external and internal sources in real time (Sporns et al., 2000). In the last decade, the identification of such connectivity pathways has become a great and debated topic in the field of basic as well as clinical neuroscience since alterations of the modes of connectivity are often associated with the pathogenesis of brain impairments (Ding et al., 2013; Kemmotsu et al., 2013; Kim et al., 2014). However, from a structural, functional, and effective point of view (Feldt et al., 2011), the common outcome is a full square connectivity matrix (CM) with *N*^2^ elements, where *N* indicates the number of considered neurons, brain regions, or assemblies. The functional connectivity matrix is relative to the correlation between time series from different sources without any underlying causal model. Correlation- and information theory-based methods are two families of algorithms used to infer functional properties (Bastos and Schoffelen, 2016). The effective connectivity matrix identifies the direct influences that one neuronal system exerts on another, relying on a network model in which different populations appear structurally connected (Lang et al., 2012). Finally, the structural connectivity matrix takes into account the physical (synaptically mediated) connections existing among neurons or small assemblies. The resolution of such CMs depends on the used technologies to acquire morphological (structural connectivity) or dynamical (functional and effective connectivity) information. By using high-density micro-electrode arrays at both *in vitro* (Simi et al., 2014) and *in vivo* (Jun et al., 2017) level, it is possible to map the neuronal position (and connections) with a single-cell resolution (tens of micrometers); on the contrary, using fMRI-based devices such a resolution is limited to small/medium brain regions (van den Heuvel and Hulshoff Pol, 2010). In any case, the CM contains different information regarding the connectivity of the considered network, from the kind of connections (i.e., excitatory vs. inhibitory links), to the synaptic weights (i.e., an indication of the synaptic efficacy), up to the delays introduced by the synaptic transmission (Fornito et al., 2016).

Once CM has been obtained, some questions arise: What are the real connections? Which are the significant ones? How many false-positive (FP) and false-negative (FN) connections are mapped inside the CM? Are weak connections significant? The answers to this batch of queries become relevant in order to keep only the relevant connections inside a CM. In other words, spurious links should be removed from the graph representing the connections of a CM. The ideal algorithm of CM thresholding should guarantee a high degree of true-positive (TP) connections, removing only the noisy ones that do not exist in the real neuronal network. With the high efficacy in keeping only the real connections, computational efficiency is also a crucial factor to consider, especially when CMs derive from large-scale neuronal networks. Many of the simplest (and fast) approaches to threshold a CM are heuristic-based and work on the detection of the weakest connections, whose removal introduces severe errors. These pruning algorithms are extremely sensible, and it is common that starting from the same CM, the application of different heuristic thresholds might achieve different thresholded connectivity matrices (TCMs). A completely different approach is based on shuffling procedures (Grun and Rotter, 2010) that allow destroying the information stored in the input signal (e.g., spike timing), obtaining independent spike trains (i.e., surrogate data). Shuffling techniques are more precise than methods based on the definition of arbitrary thresholds, but they are computationally heavy (depending on the number of generated surrogates). For this reason, it is essential to choose the best compromise between reliability and computational time.

In this work, we developed a Double Threshold (DDT) algorithm to enhance the performances of threshold-based algorithms without increasing the computational load as shuffle-based algorithms have. DDT is based on a double threshold whose fundamental aim is not to lose weak but significant connections. One of the principal drawbacks of threshold-based algorithms is that to be conservative (i.e., to not introduce many FPs), a large number of connections is arbitrarily discarded, generating graphs with a small number of connections and altered topological properties. In this work, we tested the DDT method to recover the significant connections of functional CMs obtained by applying a cross-correlation algorithm (De Blasi et al., 2019) to synthetic spike trains generated by the simulation of large-scale neuronal network models. The goodness of the DDT algorithm was evaluated comparing the TCM with the structural one, known *a priori* since part of the *in silico* model. We generated different network configurations changing the topological properties of the structural CMs. In particular, random (Erdos and Rényi, 1959), small-world (Watts and Strogatz, 1998), and scale-free (Barabási and Albert, 1999) graphs were used. Thus, we applied DDT algorithm to the functional CMs comparing its performances to the ones obtained using standard threshold-based methods, like hard threshold (HT) (Poli et al., 2015), density-based threshold (DT) (van den Heuvel et al., 2017), and a shuffling method (SH) (Kamiński et al., 2001). It is clear that the main quality for a CM thresholding method in terms of accuracy is identifying the correct size of the functional network (van Wijk et al., 2010). The DDT method highlighted how an increase in the classification accuracy led to a better quantification of the topological properties (Small-World Index and Degree Distribution) of the functional networks. Furthermore, the computational simplicity of the method ensured a computational time shorter than that required by shuffling-based approaches without losing the accuracy of link classification. Finally, we demonstrated the DDT ability to self-adapt to the size of the analyzed network: In real experimental scenarios (both *in vitro* and *in vivo*), where the real size of the network is not known *a priori* (e.g., *in silico* model), the method’s accuracy became better than the other ones, recovering the real topological characteristics of the network.

## Materials and Methods

The *ij* elements of a CM identify the strength of the relationship between nodes (i.e., neurons) *i* and *j*. Any thresholding method should keep only statistically significant connections, i.e., those due to an active connectivity between the considered nodes. In this work, we obtained CMs by means of the Total Spiking Probability Edge (TSPE) algorithm, a correlation-based method, which allows discriminating inhibitory and excitatory connections (De Blasi et al., 2019). Since *structural* connectivity properties are preserved in the *functional* CM (Bullmore and Sporns, 2009), a reliable thresholding algorithm should maintain the significant connections of the functional graph, keeping unchanged the topological properties of the structural network as well as the balance of excitatory and inhibitory links.

### The Double Threshold Algorithm (DDT)

To improve the performance of the current pruning thresholding algorithms, which consider only the strongest connections (bringing a possible incorrect description of the network’s topological properties), it was necessary to develop an adaptive algorithm that allows detecting even weak (statistically significant) connections (Schneidman et al., 2006; van Wijk et al., 2010) and that should not change the topological properties of the network for small variations of the network features (i.e., the average network’s degree).

The DDT method consists of four steps:

(1) Application of a HT to all the elements of the CM. The outcomes of this first step are two matrices: (i) the first-step thresholded connectivity matrix (T1CM) containing the strongest connections, and (ii) the rejected connectivity matrix (RM) that holds every connectivity value discarded by the application of the first threshold. The used threshold for obtaining the T1CM and the RM matrices is evaluated as the mean plus *n*-times the standard deviation of the connections’ strength.
(2) Extraction of the thresholding matrix (TM), which contains, for each *ij* element, the second thresholding values to be applied on RM. In particular:

(1) $$\left\{ \begin{array}{c} Th_{ij}^{exc}=\mu_{i,⊄ij}^{exc}+m^{exc}\cdot \sigma_{i,⊄ij}^{exc}\\ Th_{ij}^{inh}=\mu_{i,⊄ij}^{inh}-m^{inh}\cdot \sigma_{i,⊄ij}^{inh} \end{array}\right.$$

where *μ_*i*__⊄__*ij*_* and *σ_*i*__⊄__*ij*_* are the mean and the standard deviation values of the *i*th row’s non-zero-elements excluding the *ij* element, respectively, *m* is a positive arbitrary parameter. The superscripts identify the sign of the connections (excitatory or inhibitory). This step compares a single connectivity value *RM*_*ij*_ with the distribution of RM values in the *i*th row. If the *j*th and the *x*th (with *x ≠ j*) spike train are not correlated, we can think about the *x*th spike train as a “pseudo-shuffled” version of the *j*th spike train. Then, accounting for the whole *i*th row, it means that we are comparing the *ij* value with the distribution of a “pseudo-surrogate” shuffled dataset, accounting for the null hypothesis for the neuron *i*th to be functionally connected with a neuron *x*th. This is likely to be true as long as the number of spurious connection values is much larger than the number of true connection values in the *i*th row.

(3) Definition of the second thresholded matrix (T2CM) containing all the RM’s elements, which become higher than their corresponding TM’s element:

(2) $$T2CM_{ij}=RM_{ij}\text{if}RM_{ij}>TM_{ij}$$

(4) Finally, the functional connectivity matrix (FM) is defined as the union of T1CM and T2CM:

(3) FM=T1CM∪T2CM

**FIGURE 1:**
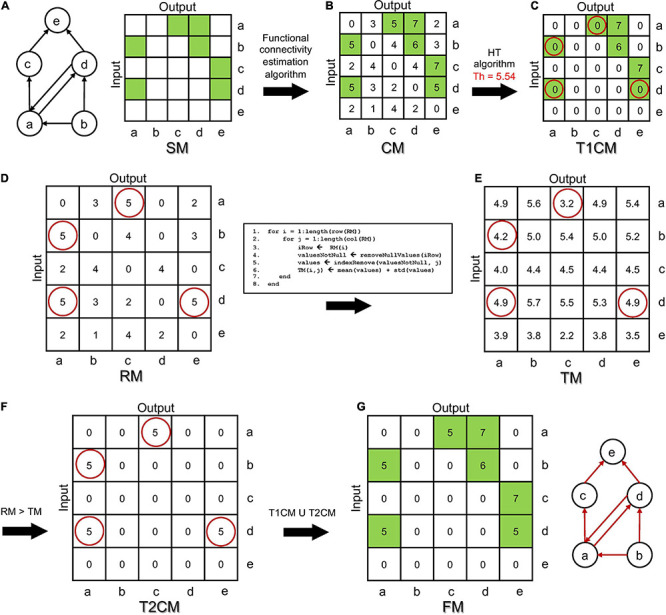
Double Threshold (DDT) algorithm operating principle applied to a simple network. **(A)** Structural graph made up of *n* = 5 nodes and the *k* = 7 structural directed links (left). Structural connectivity matrix (SM) where each green square identifies a structural connection in the graph (right). **(B)** Functional connectivity matrix (CM) obtained by applying the correlation algorithm to each pair of spike train. In the CM, the green squares show the functional connectivity values that correspond to the actually existing structural links in the network. **(C)** Applying the first hard threshold (*n* = 1), the T1CM matrix was computed. In T1CM, the red circles point to those connections incorrectly deleted from the CM (false negatives). **(D)** The rejected connectivity matrix (RM) is made of all the values that have been previously deleted by applying the hard threshold to the CM. By applying the stripping process summarized in the pseudo-code, **(E)** the thresholding matrix (TM) resulting from the application of the second step of DDT to the RM is achieved. It contains the threshold for each corresponding value of T1CM. **(F)** The T2CM shows every RM value that satisfies the condition RM > TM. **(G)** Final functional thresholded connectivity matrix (FM) and its relative functional graph.

To better understand the working principle of the DDT algorithm, the aforementioned four steps are applied to a “dummy” network made of *n* = 5 nodes (i.e., neurons) and *k* = 7 structural excitatory connections (i.e., synapses) among them (Figure 1A). The arrows identify the directionality of the connection. It is worth noticing that some connections are mono-directional (e.g., b → a), while other ones are bi-directional (e.g., a ↔ d). Each node embeds neuronal dynamics (described with the Izhikevich model), while each link incorporates a synaptic model (cf., section *“*Network Model”). We simulated 900 s of spontaneous activity, from which we inferred the CM by means of the TPSE algorithm (Figure 1B, where green squares indicate those values of the functional connections that actually correspond to the structural connections of the graph in Figure 1A). After applying the first HT (set as the mean plus one standard deviation of the non-zero elements of the CM), the T1CM is obtained (Figure 1C). This first step correctly removes all spurious values but rejects about 57% of the structural connections, i.e., false negatives (green squares with red circles, Figure 1C) that should not be discarded. In order to recover them, the RM matrix is computed (Figure 1D, left). For each *ij* element of the RM matrix (rows 1–2 of the pseudocode, Figure 1D, right), a new threshold is identified: all the non-null values of the *i*th row (rows 3–4) are identified, the *j*th value is excluded (row 5), and the threshold is computed as the average plus the standard deviation of these values (row 6). All the threshold values are collected in the TM (Figure 1E). The third step of the algorithm (Figure 1F) provides the T2CM, containing only the structural connections neglected in the first step. Finally, Figure 1G shows the final FM and the relative functional graph, which perfectly resembles the original structural one (Figure 1A).

### Selected Thresholding Algorithms for Comparison With DDT

We evaluated the performance of the DDT algorithm by comparing it with three widely employed thresholding algorithms in the literature.

The simplest, but widely used of such procedures is the HT algorithm (Poli et al., 2015), which defines a threshold on the basis of statistic distribution within the CM’s elements. In particular, considering a CM where both excitatory and inhibitory connections are present, the HT algorithm sets a threshold independently evaluated for the two kinds of connections, *Th*_*exc *_ and *Th*_*inh*_defined as:

(4) $$\left\{ \begin{array}{c} Th_{exc}=\mu_{exc}+n_{exc}\cdot \sigma_{exc}\\ Th_{inh}=\mu_{inh}-n_{inh}\cdot \sigma_{inh} \end{array}\right.$$

where μ_*exc*_ and μ_*inh*_. are the positive mean of the excitatory elements and negative mean of the inhibitory ones of the CM; σ_*exc*_ and σ_*inh*_are the standard deviations of positive and negative CM’s values, respectively. Means and standard deviations are computed over all the non-zero elements of the CM. Finally, *n*_*exc*_ and *n*_*inh*_ are two arbitrarily chosen integers.

The second considered method is the density-based threshold (DT) algorithm (van den Heuvel et al., 2017). Its working principle is based on the assumption that the network has a certain level of link density or, better, a specific *M* number of significative links. After sorting the inferred connections based on the synaptic weights, only the *M* strongest connections of the CM are taken into account. Even in this case, the algorithm consided both the *M*_*i*_ inhibitory and *M*_*e*_ excitatory strongest connections. Among the considered thresholding methods, DT is the only one that does not perform any mathematical computation of the CM’s weights. The threshold value is set in a completely arbitrary manner. Thus, in order to avoid any bias introduced by arbitrary choices and compare the DT performance with the other methods, we set the number of *M_*e*_/M_*i*_* strongest excitatory/inhibitory connections equal to the ones identified by the DDT algorithm.

The last method is based on shuffling (SH) procedures. It is completely different from both the HT and the DT, since it is based on the definition of shuffled spike trains. Taken two spike trains X and Y, we randomly shuffled the timing of each spike in the Y train, keeping constant the total number of spikes (i.e., constant MFR) but varying the interspike temporal interval (i.e., ISI). Once the Y spike train is shuffled, we computed the temporal correlation (De Blasi et al., 2019) between the X- and Y-shuffled spike trains, obtaining a null-case value of connectivity between the neuron X and Y. By iterating such operation a number of time *Nshuffling*, we were able to obtain a distribution of values that quantify the strength of the functional connectivity between X and Y while the two neurons were not functionally connected. We then compared such null-case distribution with the real connectivity values XY in the CM by a *z*-test at a certain significance level (α), assuming the null-case distribution to be normally distributed. Indeed, the SH procedure (Kamiński et al., 2001) allows achieving null-case distribution by iterating the cross-correlation estimation on different surrogate data sets obtained by shuffling the spike timings of original spike trains in order to disrupt the temporal relationship between them (Toppi et al., 2013). Although particularly accurate, SH is more time-consuming than the HT and DT methods.

The parameters of each thresholding method were kept constant for all the simulations. In particular, we set *n*_*exc*_ = 1and *n*_*inh*_ = 2 for the first step of the DDT and *m*_*exc*_ = 3 and *m*_*inh*_ = 3 for the second one. The parameters of the HT method are the same for the first step of the DDT. Finally, for the shuffling method, we set α_*exc*_ = α_*inh*_ = 0.01. The choices of the DDT parameters have been validated by sweeping their values and evaluating the number of functional links detected and the correspondent accuracy (Supplementary Figure 1) on *n* = 6 RND networks.

### Benchmarking Procedures

To evaluate the performance of the DDT algorithm and compare its results with the selected thresholding methods, we developed a large-scale neuronal network model with different topological features. In this way, we evaluated the sensibility of the DDT method to different connectivity configurations. The developed model mimicked and reproduced the typical patterns of electrophysiological activity (spiking and bursting signatures) experimentally found in dissociated cortical networks coupled to Micro-Electrode Arrays (MEAs), where connectivity spontaneously evolves following different configurations (Massobrio et al., 2015).

The use of an *in silico* model reproducing the behavior of the actual network allowed achieving a valid benchmark to test the proposed DDT algorithm since it gave the possibility to have a fully controllable system where the structural features were known *a priori*. In this way, a comparison between the functional connectivity maps obtained by thresholding the CM and the structural one can be mathematically quantified.

#### Network Model

The *in silico* model consists of a sparse network of 500 interconnected Izhikevich spiking neurons with Spike Timing Dependent Plasticity (STDP) and conduction delays (Izhikevich, 2006). We set the ratio between excitatory and inhibitory neurons at 0.8 (Marom and Shahaf, 2002). Hence, we modeled the excitatory population with 400 regular spiking neurons and the inhibitory one with 100 fast-spiking neurons (Izhikevich, 2003) according to the Izhikevich equations:

$$v^{\prime }=0.04v^{2}+5v+140-u+I$$

$$u^{\prime }=a\left(bv-u\right)$$

(5) $$\text{if}v\geq 30\text{mV},\mathrm{then}\left\{ \begin{array}{c} v\leftarrow c\\ u\leftarrow u+d \end{array}\right.$$

where *a, b, c*, and *d* are dimensionless parameters that define the neuronal type and its relative firing patterns; *v* and *u* are the neuronal membrane potential and the recovery variable, respectively. In the model, we set *a* = 0.02, *b* = 0.2, *c* = –65, and *d* = 8 for the regular spiking neurons, and *a* = 0.1, *b* = 0.2, *c* = –65, and *d* = 8 for the fast-spiking neurons. The synaptic transmission was modeled as a conduction delay between the presynaptic spike and the postsynaptic membrane potential stimulation, which was randomly assigned for each excitatory neuron in a range between 1 and 20 ms. For the inhibitory neurons, the synaptic delay was fixed at 1 ms. The network model also embedded a spike-timing dependent plasticity (STDP) model (Song et al., 2000; Caporale and Dan, 2008), where the magnitude of change of the connection weights increased as *A*_+_*e*^−*t*/τ_+_^ and decreased as *A*_−_*e*^−*t*/τ_–_^, where τ_+_ = τ_−_ = 20ms, *A*_+_ = 0.1 and *A*_−_ = 0.12 (Izhikevich, 2006).

The aforementioned neuron models were linked to define complex neuronal networks following the three canonical topologies, namely, random (RND), small-world (SW), and scale-free (SF). Independently of the topology, each network model contains 20,000 structural links, keeping the same excitatory/inhibitory neuronal ratio fixed at 0.8.

RND networks were characterized by a Poisson distribution of the node degree:

(6) $$p\left(k\right)=\frac{e^{-\delta }\delta^{k}}{k!}$$

where *k* is the mean connectivity degree of the network and *δ* is the average value of the node degree distribution (Erdos and Rényi, 1959). Random network topology was obtained interconnecting the *i*th neuron with *k* randomly chosen different neurons. Autapses were not allowed. In our implementation, we defined three modules having the same characteristics: the first one included excitatory connections linking excitatory neurons (regular spiking); the second one included excitatory connections outgoing from excitatory neurons and incoming to inhibitory ones (fast-spiking); the third one was relative to inhibitory connections going out from inhibitory neurons to excitatory ones (fast-spiking).

The SW network topology was obtained customizing the Watt–Strogatz definition (Watts and Strogatz, 1998). We generated a structural connectivity matrix made up of three different modules. First, we defined SW connections among excitatory neurons, building the “ring lattice” and then randomly redistributing a number of connections defined by the rewiring probability value, which in our simulation was set at 0.3. Second, we built SW excitatory connections from excitatory to inhibitory neurons. As long as a minimum number of strong connections in this module supported the activation of the whole inhibitory network, its presence was crucial for balancing excitation and inhibition. We randomly picked a number of connections from the ring lattice and we translated them to the connections between excitatory to inhibitory neurons. Third, we built the inhibitory module that defined the inhibitory connections generated by inhibitory neurons and that affected the excitatory ones. This module was randomly connected to the excitatory one.

Finally, SF networks were characterized by a power-law degree distribution according to Eq. (7):

(7) $$p\left(k\right)=\alpha k^{-\gamma }.$$

where γ is the characteristic exponent, which usually lies between 1.3 (slice recordings) and 3 (fMRI recordings) (Eguìluz et al., 2005; Bonifazi et al., 2009). Such distribution highlights the presence of highly connected nodes (hub neurons), which are able to influence the whole dynamics. Since the network degree is power-law stributed, we considered hubs those high-degree nodes (i.e., nodes with a degree at least one standardeviation above the average degree of the network; Sporns et al., 2007). For the aim of our analysis, we did not perform any kind of hub classification, meaning that we did not classify hubs on the basis of their participation coefficients (Sporns et al., 2007).

The SF network topology was obtained customizing the Albert–Barabasi model (Barabási and Albert, 1999). We built the SF structural connectivity matrix made up of three different modules. First, we built SF connections among excitatory neurons as defined in Barabási and Albert (1999), choosing the minimum number of SF connections per neuron. Second, we established excitatory connections from excitatory to inhibitory neurons: we defined this part of the network as a SF module with the same proprieties as the previous one. Third, we built the inhibitory module, which connected inhibitory to excitatory neurons, as another SF module with the same proprieties of the previous two. Then, since we shuffled each row of the connectivity matrix, to reduce the percentage of bi-directional connections, the SF proprieties of the network were maintained row by row. It means that the generated SF networks displayed normal incoming (i.e., accounting for the number of connections incoming a neuron) and SF outgoing (i.e., accounting for the number of connections outgoing from a neuron) degree distributions.

**FIGURE 2:**
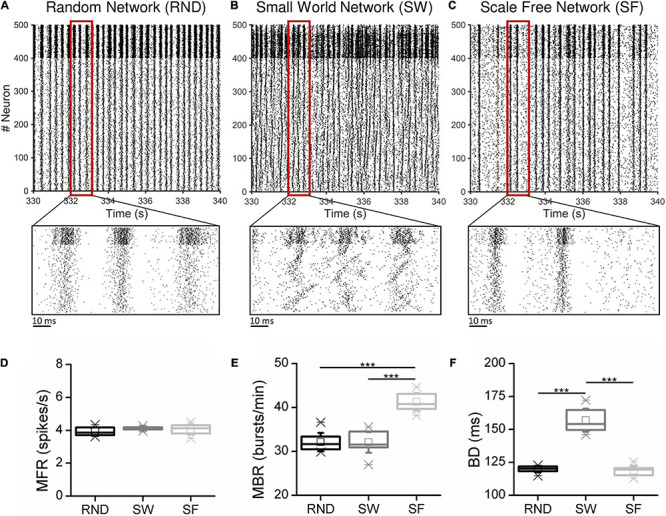
Simulated spontaneous activity of a representative **(A)** Random, **(B)** Small-World, and **(C)** Scale-Free network. The close-ups magnify 2 s of activity. **(D)** Mean firing rate (MFR), **(E)** mean bursting rate (MBR), and **(F)** burst duration (BD) averaged over 15 simulations for each topology (***p < 0.001, Kruskal–Wallis, non-parametric test).

In addition to the canonical topological connectivity rules, we also designed a network model with 500 neurons arranged in three interconnected modules (two excitatory and one inhibitory, Supplementary Figure 2A) exhibiting clearly distinct firing patterns of activity (Supplementary Figure 2B) with respect to the homogeneous configurations (Figures 2A–C). As also found in similar experimental configurations (Shein-Idelson et al., 2011; Bisio et al., 2014; Narula et al., 2017), the presence of weak connected clusters induces a decrease of the synchronization level of the network and an increase of spiking activity. This model was used as further configuration to assess the performances of the DDT algorithm.

### Performance Evaluation

#### Topological Metrics

In order to quantify the topological features of the neural networks, different quantitative metrics have been developed over the years. An exhaustive description can be found in Rubinov and Sporns (2010) and in the updated software collection Brain Connectivity Toolbox.^1^ In this work, we made use of the metrics we defined below.

**Number of links.** It is the total number of connections that define a graph (together with the number of nodes). It is the simplest feature that quantifies the network size. From the number of links, it is feasible to derive the degree distribution, which allows identifying stereotyped graphs (cf. section *“*Network Model”).

**Small-World-Index (SWI).** It is a metric that identifies the emergence of small-world properties of a network. It is computed as:

(8) $$SWI=\frac{\frac{C_{g}}{C_{rand}}}{\frac{L_{g}}{L_{rand}}}$$

where the Average Cluster Coefficient (*C*_*g*_) and the Path Length (*L*_*g*_) of the network are normalized on the expected values from random networks (*C*_*rand*_ and *L*_*rand*_, respectively) with the same number of nodes and links (Erdos and Rényi, 1959). The network exhibits SW properties if *SWI*> 1 (Humphries and Gurney, 2008). The *C*_*g*_ quantifies the network segregation and is computed as the average of all Cluster Coefficient of each node (*C*_*i*_):

(9) $$C_{i}=\frac{\#\mathrm{of}\mathrm{links}\mathrm{between}\mathrm{neighbors}\mathrm{of}i}{\frac{k_{i}\left(k_{i}-1\right)}{2}}$$

where *k*_*i*_ is the number of connections of the *i*th node. The Path Length (*L*_*g*_) identifies the integration level of the network and it is computed as the shortest distance *d*(*i,j*) between *i*th and *j*th nodes averaged over all pairs of nodes in the network:

(10) $$L_{g}=\frac{2}{N\left(N-1\right)}\sum_{i}d\left(i,j\right)$$

where *N* is the number of nodes.

#### Confusion Matrices and Accuracy Value

The confusion matrix is a tool usually adopted to solve classification problems: it compares predicted and actual values, dividing them into multiple classes if required (i.e., multi-class classification problems). Usually, the values reported in a confusion matrix are summarized in the accuracy value (ACC), defined as the fraction of correctly classified values:

(11) $$ACC=\frac{TE+TI+TN}{TE+TI+FE+FI+FN}$$

In Eq. (11), TI and TE refer to True Inhibitory and True Excitatory values (i.e., the inhibitory/excitatory connections correctly classified). FE and FI identify the false excitatory and the False Inhibitory values (i.e., the inhibitory/excitatory connections wrongly classified). Finally, TN refers to True Negative, i.e., the correct classification of the absence of connections between node pairs, and FN stands for False Negative, i.e., the existing connections not detected by the algorithm.

## Results

### Simulated Network Dynamics as Function of the Network Topologies

The network model, organized according to the RND, SW, and SF topologies described in section *“*Network Model,” was tuned up in order to generate firing dynamics reflecting the behavior of mature *in vitro* cortical cultures (Wagenaar et al., 2006). For each network topology, we simulated 15 realizations of 15 min changing: (i) the seed of the noise (modeled according to a Gaussian process) used to generate the spontaneous activity; (ii) the connections among neurons inside each network realization.

The out-degree was fixed at *k* = 40. Initial excitatory/inhibitory synaptic weights were chosen from two normal distributions (*w_*e*_* = 7, *σ_*We*_* = 1; *w_*i*_* = –7, *σ_*Wi*_* = 1). Due to the STDP evolving, the excitatory weights changed toward a bi-modal STDP distribution (Bi and Poo, 1998) after 5 min of simulation. Then, each excitatory weight was kept constant during the remaining 10 min of simulation. Figures 2A–C show 10 s of spontaneous activity (after STDP) of three representative RND, SW, and SF networks. Excitatory neurons were labeled from 1 to 400, while the inhibitory ones were labeled from 401 to 500. All the networks displayed the classical *in vitro* patterns of electrophysiological activity, i.e., high synchronized network bursts involving most of the neurons of the network, as well as bursting and spiking activity. The magnifications allow appreciating qualitatively the different modes of activation as well as the involved neuronal units. We quantified the dynamical properties of the simulated networks by means of first-order spiking/bursting metrics (Figures 2D–F). Spiking and bursting features of the simulated dataset were characterized in terms of mean firing rate (MFR), i.e., the number of spikes per second averaged over the number of the neurons of the network, mean bursting rate (MBR), i.e., the number of bursts per minute averaged over the number of the neurons of the network, and the burst duration (BD), i.e., the temporal duration of these events averaged over the entire number of detected bursts. Bursts were detected applying the string method algorithm devised in Chiappalone et al. (2005). We tuned the model parameters to achieve no significant differences in the MFR values among the three topologies (Figure 2D). Such condition was a fundamental requirement in order to ensure the comparability of the functional connectivity analysis among the different topologies. Figure 2E shows the frequency of the bursting events (we defined burst an event with three or more temporally packed spikes followed by a quiescent period longer than 100 ms). While RND and SW networks did not present statistical differences in the MBR values, with average values of 32 ± 2 and 32 ± 3 bursts/min respectively, in SF networks, higher MBR values (41 ± 3 bursts/min) were detected (*p_*SF*__–__*RND*_* = 3⋅10^−6^, *p_*SF*__–__*SW*_* = 3⋅10^−6^)^2^. Finally, the BD was affected by the clustering feature of the SW networks (Figure 2F). Indeed, this network’s topology exhibited bursts longer than those detected in RND assemblies (*p_*SW*__–__*RND*_* = 0.0004) and in SF ones (*p_*SW*__–__*SF*_* = 0.0004).

Such stereotyped patterns of electrophysiological activity were partially attenuated by simulating the electrophysiological activity of interconnected networks (Supplementary Figure 2A). From the simulations of these networks, we derived an overall MFR of 1.18 ± 0.14. spikes/s and MBR of 4.11 ± 0.68 bursts/min (Supplementary Figure 2B). It is worth to underline that it is possible to distinguish the neurons belonging to the modules from those that are not: such neurons showed a MFR of 1.78 ± 0.21 spikes/s while the neurons outside the modules displayed a MFR of 0.77 ± 0.15 spikes/s. Moreover, the neurons belonging to the modules were the only ones able to sustain bursting activity (Supplementary Figure 2B).

### Size of the Thresholded Network

**FIGURE 3:**
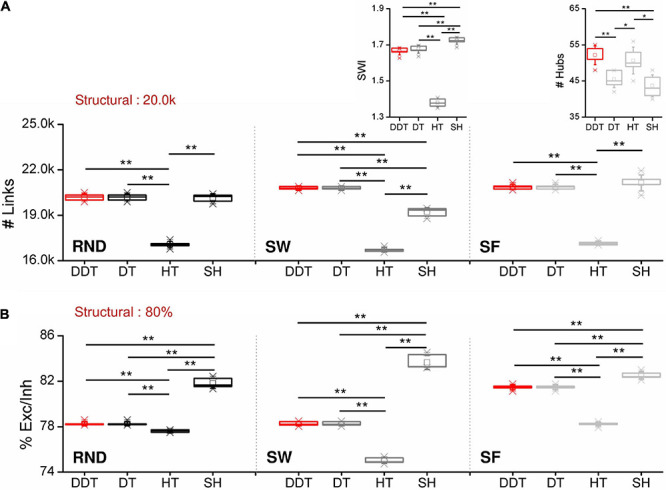
Network features as a function of the considered thresholded methods. **(A)** Number of functional links and **(B)** ratio between excitation and inhibition for RND (right), SW (middle), and SF (right) networks. The insets in the middle and right panels of **(A)** show the distributions of the SWI and of the detected hubs in SW and SF networks, respectively (*p < 0.05, **p < 0.01, Kruskal–Wallis, non-parametric test).

The network size and the physiological balance between excitation and inhibition are two of the fundamental features affecting the assessment of the network topological properties (van Wijk et al., 2010; Rubin et al., 2017). Thus, any thresholding method should identify both the right number of connections and the correct excitation/inhibition ratio. As described in section *”*Network Model” each network model contained 20,000 structural links, keeping the same excitatory/inhibitory neuronal composition (Pastore et al., 2018). All outgoing connections coming from excitatory neurons had a positive weight (therefore considered excitatory) and can target both excitatory and inhibitory neurons. On the contrary, inhibitory connections, coming from inhibitory neurons, always projected to excitatory neurons. For the following analysis, *n* = 6 realizations for each topology were considered. First, we evaluated and compared the total number of links (Figure 3A) and the ratio between excitatory and inhibitory links (Figure 3B) identified by the four thresholding methods applied to the three topologies (RND, SW, and SF). Since the DT method sets a threshold value depending on the desired density to achieve, to compare without any kind of bias DT with the other three methods, we forced DT to detect the same number of links of DDT as described in section *“*Selected Thresholding Algorithms for Comparison With DDT”. For this reason, the number of links (Figure 3A) obtained by the DDT and DT methods were exactly the same.

In RND networks, the SH method provided the same number of functional links identified by the DDT one (*p*_*DDT–SH*_ = 0.323). The DDT identified 20,377 ± 138 links, close to the structural target set at 20,000 connections. In this topological organization, only HT algorithm underestimated the number of links by detecting 17,095 ± 92 connections, significantly lower than DDT (*p*_*DDT*−*HT*_ = 0.004).

In SW networks, the SH method underestimated the number of functional links with respect to DDT (*p* = 0.004), while DDT detected the highest value of links 20,794 ± 97. As in RND networks, HT neglected about 16% of significant links. Given the intrinsic structural “small-world” properties, we evaluated the SWI (Eq. 8) of the inferred functional networks, and we compared it with the structural target (Figure 3A, middle, inset). All the thresholding methods allowed achieving SWI values greater than 1, meaning that reconstructed functional networks maintained the small-worldness properties. By analyzing the SWI values of the single methods, we found that SH showed the highest value (1.73 ± 0.01). Nevertheless, the DDT guaranteed a SWI = 1.67 ± 0.02, different but close to SH (*p*_*DDT–SH*_ = 0.004) and significantly higher than HT, which showed a SWI = 1.37 ± 0.02 (*p*_*DDT–HT*_ = 0.004). Since the structural SW networks showed SWI = 1.83 ± 0.01, we demonstrated that the weak connections recovered by the DDT algorithm contribute to bring the SW properties of the functional networks closer to those of structural ones.

Also in SF networks, the DDT detected a number of connections comparable to the SH method and significantly higher than HT (*p*_*DDT*−*HT*_ 0.004). The key feature of SF networks is the existence of hub neurons. We evaluated how the number of hubs was influenced by the thresholded methods (Figure 3A, right, inset). The number of hub neurons computed over all the SF structural matrices was 49 ± 2. The implementation of the DDT algorithm allowed detecting 52 ± 2. Significant differences were observed between DDT and DT (*p*_*DDT*−*DT*_ = 0.004)and SH methods (*p*_*DDT*−*SH*_ = 0.003), suggesting that the weaker links recovered by DDT were non-uniformly distributed within SF networks, but, on the contrary, made a greater contribution to the hub growth.

The excitatory/inhibitory ratio (Figure 3B) detected by the DDT method was higher than HT in every considered topology [*p*_*DDT–HT  (RND)*_ = 0.006, *p*_*DDT–HT  (SW)*_ = 0.004, *p*_*DDT–HT  (SF)*_ = 0.004], lying in between HT and SH. Overall, the four considered methods were able to detect a percentage of excitation close to the structural target of 80%.

The same investigations were performed on the modular networks shown in Supplementary Figure 2. In this type of network, the HT method detects a number of connections close to the structural model (Supplementary Figure 2C). Under these conditions, the number of connections recovered by DDT is consistently lower than in RND, SW, and SF networks and does not change the accuracy of the link classification (section “Accuracy and Computational Time”). Also, in modular networks, the excitation/inhibition ratio is very close to the structural level of 80% (Supplementary Figure 2B) and there are no statistical differences between the various methods.

### Degree Distribution of the Networks

**FIGURE 4:**
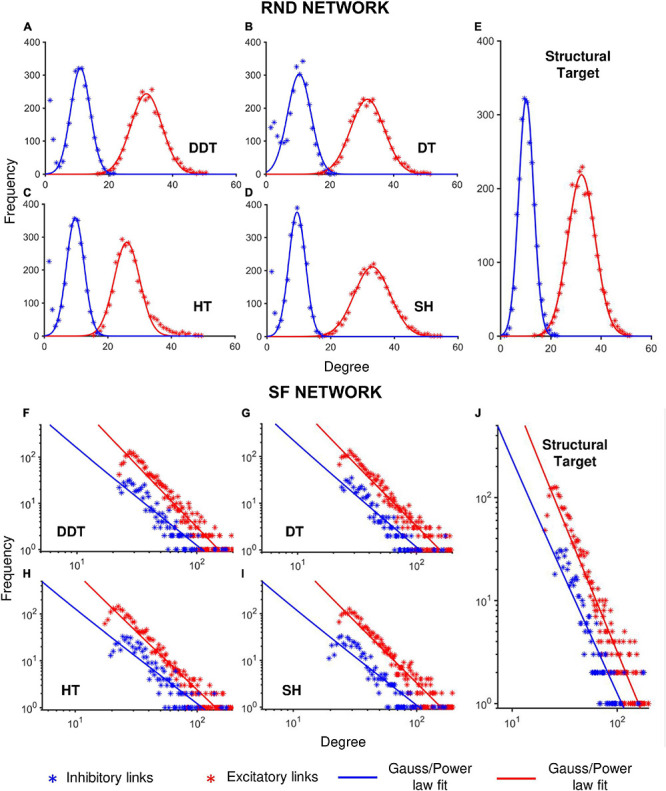
Degree distribution of RND and SF networks as a function of the different thresholding methods. Each panel shows the cumulative degree distributions evaluated over *n* = 6 simulations, considering separately the excitatory (red) and inhibitory (blue) subnetworks. **(A–D)** Degree distributions of RND networks thresholded with DDT, DT, HT and SH methods, respectively. **(E)** Structural degree distribution of the randomly connected excitatory and inhibitory sub-populations. **(F–I)** Degree distribution of SF networks thresholded by DDT, DT, HT, and SH methods, respectively. **(J)** Structural degree distribution of the SF excitatory and inhibitory sub-populations. *p < 0.05, **p < 0.01.

While topological features of SW networks can be quantified by the computation of the SWI (section “Network Model”), RND and SF topologies are identified by their degree distributions, which should resemble normal and power-law relationships, respectively (section “Network Model”). Thus, we verified the capability of DDT (and the other methods) to recover the structural degree distribution of the simulated RND and SF networks from the thresholded functional connectivity matrices. Figures 4A–D,F–I show the cumulative degree distribution of *n* = 6 RND and SF networks, respectively, by splitting the excitatory (red) and inhibitory (blue) sub-populations and the different thresholding algorithms. The structural degree distributions (target) of both RND (normal distribution, $R_{exc}^{2}$ = 0.99;$R_{inh}^{2}$ = 0.98) and SF (power-law distribution, $R_{exc}^{2}$ = 0.90;$R_{inh}^{2}$ = 0.78) networks are reported in Figures 4E,J. For all the thresholding methods, the goodness of the Gaussian fit showed significant values for the excitatory subnetworks (*R*^2^ greater than 0.97), while for the inhibitory one, the accuracy of the fit spanned from 0.76 for the DDT to 0.84 for the SH (Table 1). Such a result could be explained by the smaller number of inhibitory neurons within each RND network, which makes a good reconstruction of the distribution more difficult.

**TABLE 1:** Coefficients of determination of the excitatory (**$R_{\mathrm{exc}}^{2})$** and inhibitory (**$R_{\mathrm{inh}}^{2})$** gaussian models used to fit RND networks degree distribution.

***Coefficient of determination (RND)***	***DDT***	***DT***	***HT***	***SH***
*$R_{exc}^{2}$*	0.99	0.99	0.97	0.99
*$R_{inh}^{2}$*	0.76	0.79	0.77	0.84

From the fitting curves, we estimated the mean and the standard deviation of the Gaussian curves (Table 2), which were comparable with the structural degree distribution of excitatory (30.9 ± 7.2) and inhibitory (10.1 ± 4.6) connections.

**TABLE 2:** Mean and standard deviation of the fitting gaussian curve.

***Mean ± std Gaussian distribution***	***Structural target***	***DDT***	***DT***	***HT***	***SH***
*Exc population*	30.9 ± 7.2	31.8 ± 8.3	31.8 ± 8.1	26.0 ± 5.4	33.0 ± 9.6
*Inh population*	10.1 ± 4.6	11.0 ± 5.2	10.3 ± 5.1	9.78 ± 4.0	9.5 ± 3.9

As previously pointed out (Figure 3A), the HT method underestimated the size of the networks and consequently the average values of the distribution of the number of connections of each single neuron (26.0 ± 5 for excitatory neurons; 9.78 ± 4.0 for the inhibitory ones).

The same analyses and comparisons were performed for SF networks too (Tables 3, 4). In this configuration, the goodness of the fit derived from the four methods was almost equivalent (Table 3). Overall, the coefficients of determination were lower than those of the RND topology, indicating that the SF properties were more difficult to recover.

**TABLE 3:** Coefficients of determination of the excitatory (**$R_{\mathrm{exc}}^{2})$** and inhibitory (**$R_{\mathrm{inh}}^{2})$** power-law models used to fit SF network degree distribution.

***Coefficient of determination (SF)***	***DDT***	***DT***	***HT***	***SH***
*$R_{exc}^{2}$*	0.88	0.88	0.90	0.89
*$R_{inh}^{2}$*	0.80	0.81	0.76	0.80

**TABLE 4:** Slope of the fitting power law for SF network degree distrinution using the different thresholding methods.

***Slope of fitting curve (SF)***	***Structural target***	***DDT***	***DT***	***HT***	***SH***
**Slope*_*exc*_*	–2.5	–2.6	–2.6	–2.5	–2.6
**Slope*_*inh*_*	–2.2	–2.1	–2.2	–2.0	–2.0

Power-law fitting curves were evaluated in terms of their slopes and compared to the structural ones (Table 4). For each thresholding method, we observed greater slope (absolute) values of the excitatory subnetworks than of the inhibitory ones in accordance with the structural fitting. Numerically, all the thresholding methods allowed achieving closed value to the structural degree distributions (which presented a slope of –2.5 and –2.2 for excitatory and inhibitory neurons, respectively, Table 4). These results suggested how the structural properties of SF networks were well coded also in the correspondent functional networks.

### Accuracy and Computational Time

In order to evaluate the performances and the accuracy of the four thresholding methods, we evaluated the confusion matrices of the different network topologies.

**FIGURE 5:**
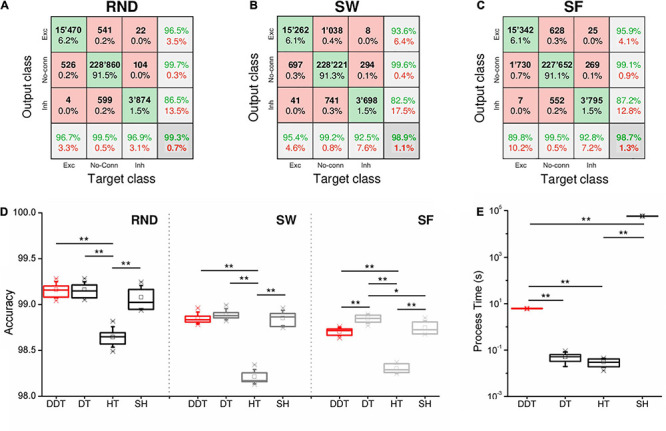
Accuracy of the thresholding methods. Three examples of confusion matrices obtained with the DDT algorithm for **(A)** RND, **(B)** SW, and **(C)** SF topologies. The target and the output classes have been divided in excitatory (Exc), absent (No-Conn), and inhibitory (Inh) connections. Green/red squares indicate correct/incorrect number/percentage of classified links. **(D)** Accuracy evaluated through confusion matrices over all kind of topologies and thresholding algorithms. **(E)** Computational time requested by each method to threshold *n* = 6 CM of RND networks (*p < 0.05, ** *p* < 0.01, Kruskal–Wallis, non-parametric test).

Each confusion matrix shows the classification over three classes (i.e., excitatory connections, inhibitory connections, or non-connection) of the links identified by the DDT. The “output class” refers to the class in which the thresholding algorithm classified each link, while the “target class” refers to the class of each considered structural link. Looking at the fractions of correctly/incorrectly classified connections in the confusion matrices (green/red squares), we were able to compare the accuracy of the DDT method in the three topologies. Figures 5A–C show three confusion matrices relative to one simulation of each network topology whose CM was thresholded by the DDT algorithm. RND topology presented the lowest percentage of misclassified connections (0.7%), confirming to be the easiest topology to recover. Further on, the classification of links in the SW and SF networks showed a different trend. In the SW assemblies, we estimated a greater number of false excitatory (FE) than false-negative (FN) samples, while the opposite scenario occurred in SF networks. Referring to the SW examples reported in Figure 5B, 1,038 connections (0.4%) were classified as FE, when they actually are non-existent connections at a structural level. On the contrary in the SF network of Figure 5C, the DDT method classified as FN 1,730 excitatory connections (0.7%) that actually are excitatory structural connections. The same observations can be made about inhibitory links. Since the DDT parameters did not change over the topologies, such behavior was only affected by the way in which the connection weights were distributed within the two network types.

The accuracy of RND, SW, and SF networks is compared in Figure 5D. For all the topologies, no significant differences in the accuracy between DDT and SH were observed. In each topology, both DDT and SH outperformed the accuracy achieved with the HT (*p*_*DDT–HT*_ = 0.004, *p*_*SH–HT*_ = 0.004). It is worth noticing that in SF networks, DT results are more accurate than DDT (*p* = 0.006): such a result can be partially explained by the presence of hubs that introduced a lack of homogeneity in the distribution of the connectivity, on which the statistics underlying the HT and DDT methods were based. The modular configuration and a lower firing rate of the network led to a more difficult identification of the conntions. Indeed, the DDT showed an overall accuracy level of 0.971 ± 0.001 (Supplementary Figure 2E), and there are no statistical differences with any of the other methods. In our opinion, this may be due to two main factors: firstly, the starting point of our analysis was the algorithm of cross-correlation that allowed inferring the measure of connectivity. These kinds of correlation-based algorithms suffer low values of firing rate (Aertsen and Gerstein, 1985; Aertsen and Gerstein, 1991). Thus, modular networks could display dynamics more difficult to encode in terms of functional connectivity, leading to a less accurate non-thresholded CMs. Secondly, we explored a case where the first DDT threshold (i.e., the HT) already identified a number of connections close to the structural target. Even though the second threshold added other links to the first thresholded network, this addition: (i) added few links with respect to the RND, SW, and SF networks (this can be appreciated by looking at the differential between the number of links detected by DDT and HT); and (ii) did not affect the overall accuracy of the thresholding in a case where there is no need.

Finally, the computational effort requested by the methods was computed. All the tests were carried out on an Intel Xeon 2.7 GHz, RAM 64 GB, on a subset of *n* = 6 random networks. The DT and HT methods could be considered instantaneous methods, since they threshold a CM in 0.03 ± 0.01 s and 0.05 ± 0.02 s, respectively. On the other hand, about 16 ± 1 h were required to perform the same operation with SH (*p*_*SH–DT*_ = 0.004, *p*_*SH–HT*_ = 0.004). Considering the performances obtained with the DDT method (Figures 3, 5D), its process time is indeed a good compromise: It reduces the time cost of the SH of about 99% (*p*_*DDT–SH*_ = 0.004) (Figure 5E). Thus, the DDT and DT algorithms became the two methods that guarantee better performances and lower process time.

To prove the advantage of the DDT algorithm in the reconstruction of neuronal networks with respect to DT, we evaluated the stability and reliability of the two methods under conditions similar to the experimental ones. This allows examining networks where the topology is not known *a priori* or that present a mixture of topological properes such as hubs and clustered ensembles, defining SF networks with SW attributes (Pastore et al., 2018). The presence of unknown different configurations makes it harder to identify the right number of structural links (especially in the case of highly connected networks), as well as the synaptic efficacy (synaptic strength).

**FIGURE 6:**
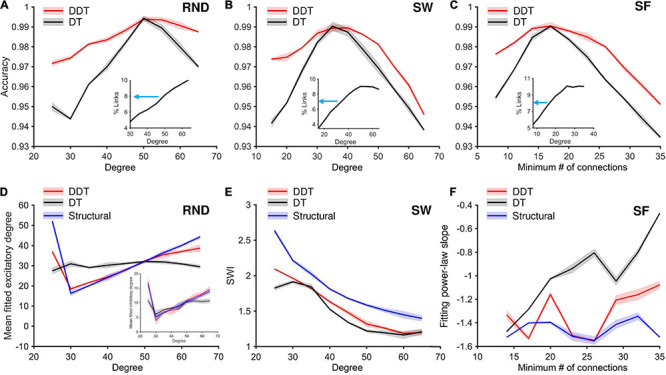
Accuracy analysis of DDT and DT algorithms by sweeping the mean degree of RND and SW networks, and the minimum number of connections for SF networks. **(A–C)** The trend of the link classification accuracy values in RND, SW, and SF matrices, respectively, calculated by applying the DDT (red line) and DT (black line) methods. For each value of *k*, three networks were simulated, for each of the different topologies. In each of these panels, the trend of the density of the structural networks (in the form of percentages of links actually existing in the network with respect to the total possible number of connections) is represented as an inset, increasing the average degree *k* in the RND and SW networks, the minimum number of connections *k* for each neuron in the SF networks. The blue arrows indicate the median value of the density curve, which is used to define the *M*_*i*_ and *M*_*e*_ parameters by applying the DT method. **(D)** Mean value of the fitted excitatory normal degree distribution computed across all networks by varying the structural degree *k*, comparing networks thresholded with DDT (red line), DT (black line), and the structural target (blue line). The inset shows the fitted inhibitory degree. **(E)** SWI of SW networks by varying the structural degree *k*, comparing networks thresholded with DDT (red line), those with DT (black line), and the structural target (blue line). **(F)** Slope of the fitted total (i.e., both excitatory and inhibitory links) power-law degree distribution computed across all networks by varying the structural degree *k*, comparing networks thresholded with DDT (red line), those with DT (black line), and the structural target (blue line).

In addition, we analyzed the behavior of DT and DDT fixing the parameters of both methods and varying the network degree. For the DT, we fixed *M*_*i*_ and *M*_*e*_ to match the percentage of existing links (i.e., density) with the median value of density curves over all the variation of the degree *k* (Figures 6A–C, insets). We set to a final density values of 8% in the RND and SF networks and 7% in the SW networks. The accuracy of DDT and DT was evaluated over 90 simulated RND (Figure 6A), SW (Figure 6B), and SF (Figure 6C) networks. The DDT accuracy curve (red line) in the RND network was always above the one obtned with DT (black line), except at *k* = 50 (Figure 6A). For such a degree, where the maximum accuracy values were obtained, the density of the structural network (8%) is exactly equal to the median value indicated by the blue arrow in the inset and used as a threshold in the DT method. The outcome highlighted the effectiveness of the DT only if the number of connections is known exactly beforehand. As soon as this information was missing, the reliability of this method collapses, as its accuracy does. On the contrary, the DDT method maintained a minimum accuracy of 0.97 over the entire range of *k* variation. The same considerations were also preserved in SW (Figure 6B) and SF (Figure 6C) networks, although we should observe a more pronounced fall in the accuracy for both DDT and DT methods. This trend could probably be due to the nature of the structural connectivity of the SW and SF networks, which makes their reconstruction from functional networks more difficult.

The previous analysis proved that a crucial feature of a thresholding method is the capability of inferring the number of connections that define the real structural network and to recover a functional network with a comparable size. The accuracy of the DDT and DT methods was pretty similar when they were forced to detect the same number of links (intersection between black and red curves of Figures 6A–C). When the assumed network density was different from the structural density of the network, the two curves diverged (Figure 6). An excessive number of missing links may lead to incorrect quantification of the network size in the case of RND networks, and to an underestimation of SWI values in SW networks. Similarly, an excess of links may lead to a rapid fall in the accuracy (Figure 6A). Considering the RND realizations, if network density was overestimated of about 2% (8% in the functional vs. 6% in the structural network), the accuracy of DT thresholding dropped from 0.99 to about 0.97. In the same condition, DDT provided a slower fall in accuracy than DT (*p* = 0.008).

Finally, we proved how the lack of accuracy led to a significant variation of the topological properties of the networks. From the same RND, SW, and SF networks used to analyze the accuracy of the DDT and DT methods as a function of the degree *k*, we evaluated the mean value of the normal excitatory degree distribution in the RND (Figure 6D, inhibitory degree distribution as inset), the SWI for the SW (Figure 6E), and the slope of the power-law fitting of the SF networks (Figure 6F). The CM of RND networks thresholded with the DDT algorithm displayed an average value of the normal fitting distribution statistically equivalent to the structural ones (Figure 6D, blue curve) for *k* = [30, 60], both for the excitatory (Figure 6D, red curve) and inhibitory links (Figure 6D, red curve, inset). This means by sweeping the degree *k*, and therefore the number of connections within the functional RND networks, the DDT method managed to adapt the number of connections recovered to maximize the accuracy and reconstruct the RND feature.

On the other hand, the DT method (Figure 6D, black curve) kept a constant average degree for all the considered range, without following the increase in the number of excitatory connections of the structural networks. Such behavior was not observed in the fitting of the inhibitory degree (inset of Figure 6D). For the entire range of variation of *k*, the CM thresholded by DDT and DT showed average values of inhibitory degree not different from each other, and statistically equal to the structural target highlighted in blue.

A similar trend was found for SW networks (Figure 6E), where the SWI obtained through DDT showed a trend (red line) resembling the structural one (blue line) but shifted down since structural and functional networks are only partially correlated (Straathof et al., 2019). On the contrary, the SWI profile achieved by the DT method (blue line) displayed values statistically lower (*p* < 0.05) than both the structural networks and the ones obtained with DDT for all the considered points of the graph, except for the *k* values that correspond to the DT accuracy peak (*k* = 35 and 40 for the structural and functional networks, respectively).

Finally, the use of DT algorithm in SF networks induced a drop in accuracy and led to a marked error in quantifying the slope of the degree fitting distribution (Figure 6F). For all *k* values, the slope of the power-law fitting obtained in the networks stripped with the DDT method (red line) remained closer to the structural target curve (blue line) than that obtained with the DT method (black line).

The same investigation was performed on the modular networks described in (section “Network Model”). By spanning the degree of such networks within the interval of *k* = [60, 95], the overall accuracy of the link classification never reaches 98% with either DDT or DT (Supplementary Figure 2E). By setting the DT threshold parameters in order to achieve a network density of 6.5% (Supplementary Figure 2E, inset), a statistical difference in accuracy between DDT and DT can be seen for *k* = [60, 70] (*p* = 0.008).

## Discussion and Conclusion

In the last years, several attempts have been performed to develop computational approaches and algorithms able to derive functional connectivity matrices from different kinds of time series deriving from the recording of electrophysiological activity (Ito et al., 2011; van Bussel et al., 2011; Poli et al., 2016; Pastore et al., 2018). The boost that also technological efforts gave in developing new powerful devices able to acquire both at *in vitro* and *in vivo* level a huge number of units allowed to manage large connectivity matrices mapping large-scale neuronal ensembles (Simi et al., 2014; Jun et al., 2017). However, no universal standard methodologies exist to threshold such connectivity matrices in order to discharge unfruitful connections. The necessity to find a high-performing method comes from the fact that this operation of selection of the significant connections can dramatically modify all the analyses regarding the topological properties of the network. To this end, we developed and tested a method that brings together the merits of some of the currently used approaches but increasing the accuracy as well as the computational load. To test the DDT method and compare with a selection of state-of-the-art algorithms, we developed *in silico* large-scale neuronal networks, with different topologies, namely, random, small-world, and scale-free, in order to mimic the electrophysiological patterns of spontaneous activity of cortical *in vitro* assemblies.

The DDT algorithm ensured a better estimation of the size of functional networks, in terms of number of links and the ratio between excitatory and inhibitory links, leading to better accuracy of link classification and a better transition from structural to functional networks.

The DDT method allowed the detection of a number of links comparable to the ones provided by the SH (and compatible with the structural target) but reduced the computational time by more than 99%. Also, the accuracy of the DDT method evaluated over the different topologies (Figure 5D) highlights its goodness (together with DT and SH) to identify the right (i.e., functional with a structural counterpart) connections. However, it is worth noticing that DT and DDT are able to generate a thresholded CM in a quasi-real-time fashion, differently from SH, which requires tens of hours. In this sense, DDT and DT are the best candidates to efficiently and effectively threshold a CM. However, the application of DT, unfortunately, involves a completely arbitrary choice of the size of the network that is obtained by this method. When this choice turns out to be largely outside the true size of the network one wants to reconstruct, the results can lead to networks whose features are very far from the real ones. On the other hand, thanks to two successive thresholding steps, the DDT manages to be a good compromise between the complete arbitrariness of simpler thresholding methods and the statistical significance of shuffling-based methods.

## Data Availability Statement

The network model files (Matlab) and the customized functions (Matlab) used to analyze the data have been deposited in Zenodo. The DOI of the code reported in this article is https://doi.org/10.5281/zenodo.5085423.

## Author Contributions

AB developed the DDT algorithm and ran analyses and simulations. MB supported the development of the method, contributed to the data analysis, and prepared the figures of the manuscript. PM developed the computational model and supervised the work. All authors wrote the manuscript.

## Conflict of Interest

The authors declare that the research was conducted in the absence of any commercial or financial relationships that could be construed as a potential conflict of interest.

## Publisher’s Note

All claims expressed in this article are solely those of the authors and do not necessarily represent those of their affiliated organizations, or those of the publisher, the editors and the reviewers. Any product that may be evaluated in this article, or claim that may be made by its manufacturer, is not guaranteed or endorsed by the publisher.

## Footnotes

1
https://sites.google.com/site/bctnet/

2 Since data do not follow a normal distribution (Kolmogorov–Smirnov normality test), the non-parametric Kruskal–Wallis test was applied. Significance levels were set at *p* < 0.05.
