# Supplementary material for: Thresholding Functional Connectivity Matrices to Recover the Topological Properties of Large-Scale Neuronal Networks
Source: Front Neurosci. 2021 Aug 16;15:705103. doi: 10.3389/fnins.2021.705103 (PMC8415479; doi:10.3389/fnins.2021.705103)
Supplement: Supplementary file 1 [file Data_Sheet_1.PDF]

SUPPLEMENTARY MATERIALS

Thresholding functional connectivity matrices to recover the topological properties of large-scale neuronal networks

Alessio Boschi[1,*], Martina Brofiga[1,*], Paolo Massobrio[1,2]

[1] Department of Informatics, Bioengineering, Robotics, System Engineering (DIBRIS), University of Genova, Genova - Italy.

[2]National Institute for Nuclear Physics (INFN), Genova, Italy

[*]These authors contributed equally to this work

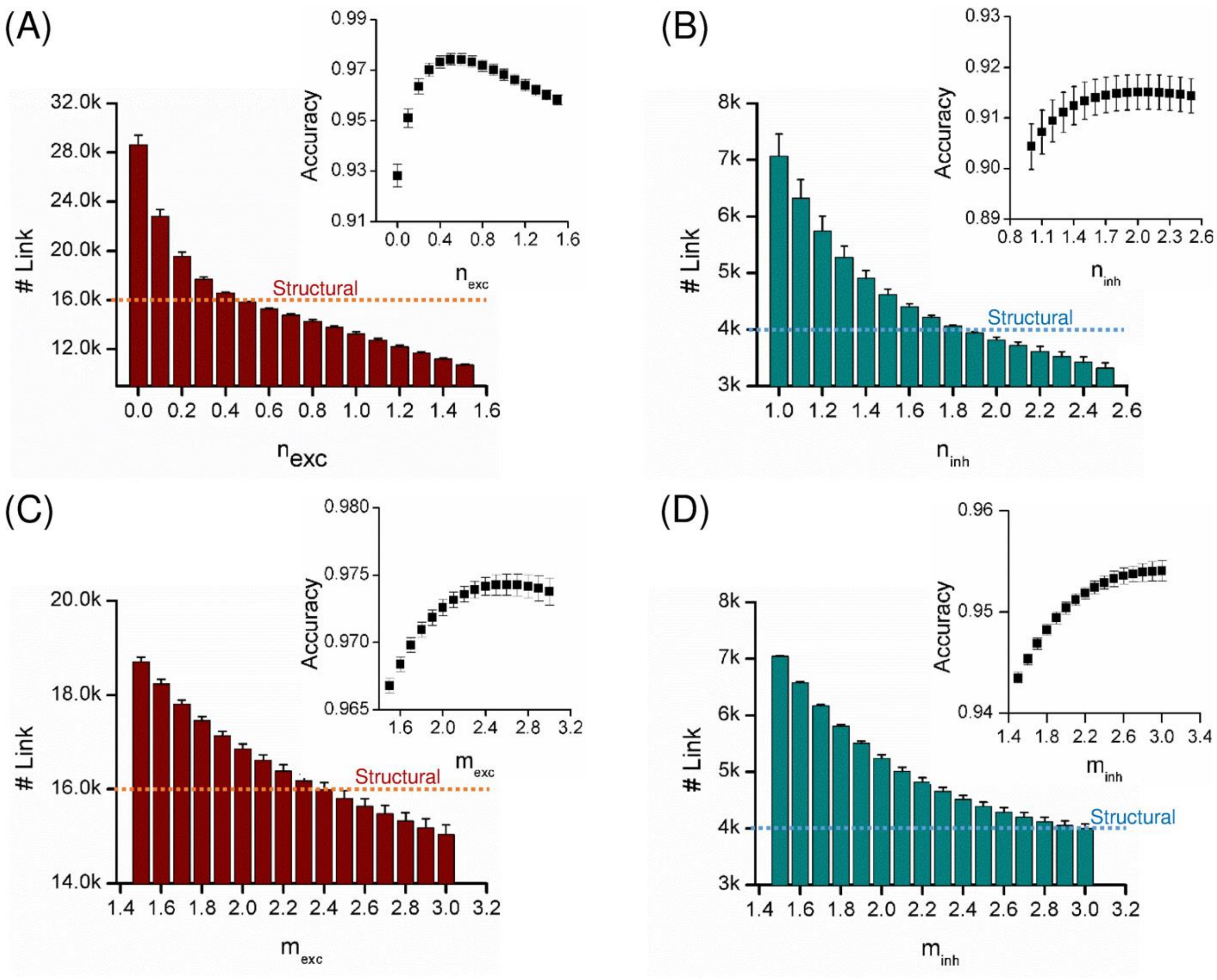

Figure S1| DDT parameters identification. DDT algorithm applied to 20 SF matrices by sweeping the (A) n_{exc}, (B) n_{inh}, (C) m_{exc} and (D) m_{inh} parameters. The chosen values ensure a number of detected links close to the structural target, as well as a fraction of well-classified links (inset) greater than 0.8.

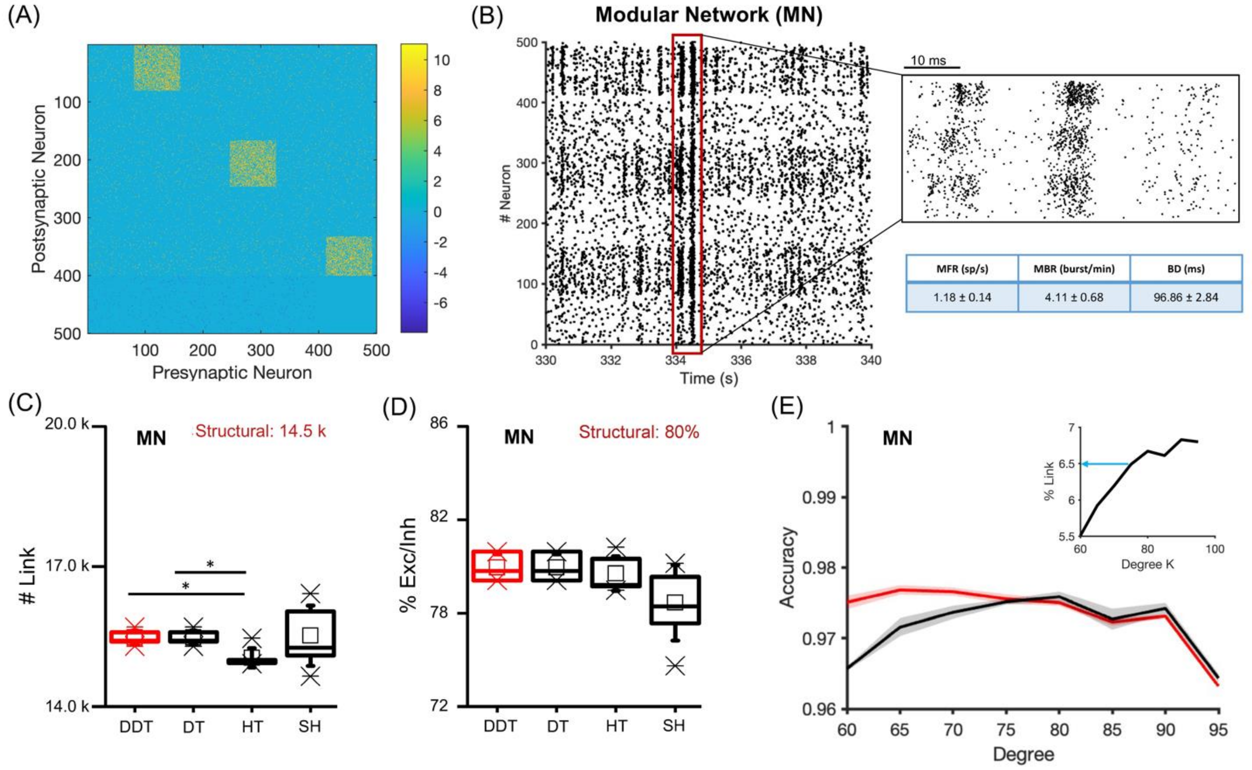

Figure S2 | Simulated spontaneous activity of a representative modular network. (A) Structural connectivity matrix. (B) 10-second electrophysiological activity with a close-up of 1 s of activity. The Table summarizes the Mean Firing Rate (MFR), Mean Bursting Rate (MBR), and burst duration (BD). (C) Number of functional links and (D) ratio between excitation and inhibition for modular networks. (E) Trend of the link classification accuracy values in modular networks, calculated by applying the DDT (red line) and DT (black line) methods. For each value of degree, 3 networks were simulated. The trend of the density of the structural networks (in the form of percentages of links actually existing in the network with respect to the total possible number of connections) is represented as an inset, increasing the average degree k. The blue arrows indicate the median value of the density curve, which is used to define the M_i and M_e parameters by applying the DT method. Data are averaged over a dataset of n = 6 simulations. (* $p < 0.05$, Kruskal-Wallis, non-parametric test).
